# Somitic mesoderm morphogenesis is necessary for neural tube closure during Xenopus development

**DOI:** 10.3389/fcell.2022.1091629

**Published:** 2023-01-09

**Authors:** Neophytos Christodoulou, Paris A. Skourides

**Affiliations:** Department of Biological Sciences, University of Cyprus, Nicosia, Cyprus

**Keywords:** neural tube defects, embryogenesis, apical constriction, neural tube closure, Xenopus

## Abstract

Neural tube closure is a fundamental process during vertebrate embryogenesis, which leads to the formation of the central nervous system. Defective neural tube closure leads to neural tube defects which are some of the most common human birth defects. While the intrinsic morphogenetic events shaping the neuroepithelium have been studied extensively, how tissues mechanically coupled with the neural plate influence neural tube closure remains poorly understood. Here, using *Xenopus laevis* embryos, live imaging in combination with loss of function experiments and morphometric analysis of fixed samples we explore the reciprocal mechanical communication between the neural plate and the somitic mesoderm and its impact on tissue morphogenesis. We show that although somitic mesoderm convergent extension occurs independently from neural plate morphogenesis neural tube closure depends on somitic mesoderm morphogenesis. Specifically, impaired somitic mesoderm remodelling results in defective apical constriction within the neuroepithelium and failure of neural tube closure. Last, our data reveal that mild abnormalities in somitic mesoderm and neural plate morphogenesis have a synergistic effect during neurulation, leading to severe neural tube closure defects. Overall, our data reveal that defective morphogenesis of tissues mechanically coupled with the neural plate can not only drastically exacerbate mild neural tube defects that may arise from abnormalities within the neural tissue but can also elicit neural tube defects even when the neural plate is itself free of inherent defects.

## Introduction

The central nervous system is the processing center of all vertebrate animals, controlling sensation, movement, emotions, communication, responses, thought processing, and memory. Thus, correct central nervous system formation during embryogenesis is central both for embryo survival and adult life quality. The first critical event during central nervous system development is the formation of the neural tube, the precursor of the central nervous system (Nikolopoulou et al., 2017). The neural tube emerges from the dorsal ectoderm and gives rise to the brain (anterior neural tube) and the spinal cord (posterior neural tube) (Wallingford, 2005). Defective neural tube formation leads to neural tube defects which are one of the most common human birth defects, occurring in .5–2 per 1000 births, affecting 324,000 infants per year, leading to 88,000 deaths and 8.6 million disability-adjusted life years (Greene and Copp, 2014; Zaganjor et al., 2016). Thus, understanding the molecular and cellular processes underlying neural tube formation will lead to a better understanding of human neural tube defects and facilitate their prevention.

The transformation of the flat neuroepithelium to the neural tube is primarily driven by forces generated by intrinsic morphogenetic events. Convergent extension and apical constriction are the two main morphogenetic movements taking place within the neuroepithelium during neural tube closure (Keller et al., 1992; Davidson and Keller, 1999; Hildebrand and Soriano, 1999; Haigo et al., 2003; Nishimura et al., 2012; Zanardelli et al., 2013; Christodoulou and Skourides, 2015; Baldwin et al., 2022). In addition to neural plate generated forces, it has been suggested that extrinsic forces stemming from the adjacent surface ectoderm and the underlying somitic mesoderm actively contribute to neural tube closure (Schroeder, 1970; Karfunkel, 1974; Jacobson and Gordon, 1976; Moury and Schoenwolf, 1995). Recently, we showed that the surface ectoderm does not actively contribute to neural tube closure, but its proper development is permissive for proper neural plate morphogenesis (Christodoulou and Skourides, 2022). However, the role of somitic mesoderm morphogenesis during neural tube closure is not well defined.

During neural tube closure the somitic mesoderm, which underlies the neural plate, undergoes convergent extension (Wilson et al., 1989; Keller and Sutherland, 2020). During this process, the somitic mesoderm elongates along the embryo anteroposterior axis, becomes thinner along the mediolateral axis, and in addition expands along the dorsoventral axis. Mouse knockout embryos for PCP genes display neural tube defects due to defective convergent extension of the neuroepithelium. However, at the same time such mutants display defects in somitic mesoderm convergent extension (Lu et al., 2004; Yen et al., 2009). While the contribution of neural plate convergent extension to neural tube closure has been clearly demonstrated (Wallingford and Harland, 2001), the contribution of somitic mesoderm convergent extension to neural tube closure has not been addressed directly.

Movement of the underlying mesoderm during zebrafish gastrulation, has been shown to generate friction forces, affecting the movement of the abutting ectoderm (Smutny et al., 2017). Thus, it is possible that at neurula stages, the somitic mesoderm is generating and imposing forces on the neural plate. Such forces could be required directly to actively shape the neuroepithelium through force transmission but could also be needed indirectly by stimulating mechanosensitive signaling cascades within the neural plate. In both scenarios somitic mesoderm morphogenesis actively contributes to neural tube closure. On the other hand, it is plausible that somitic mesoderm morphogenesis does not have an active role during neural tube closure but similarly to the surface ectoderm, its morphogenesis may be permissive for the neural tube formation. In this scenario, defects in medial thinning and dorsoventral expansion in the paraxial mesoderm could result in generation of resistive forces which the neuroepithelium will have to overcome during neural tube formation.

Here, we aim to elucidate the contribution of somitic mesoderm morphogenesis during neural tube closure. To achieve this, we abrogated somitic mesoderm convergent extension in Xenopus embryos. Our data show that inhibition of somitic mesoderm convergent extension by downregulation of the somitic mesoderm specific cell cycle regulator Wee (Leise and Mueller, 2004), is accompanied by defective neural tube closure. Furthermore, we show that somitic mesoderm convergent extension is essential for apical constriction and hinge point formation. Last, we show that mild defects in neural plate and somitic mesoderm morphogenesis have additive effects on neural tube closure, resulting in severe neural tube defects. Overall, our data show that somitic mesoderm restricted defects can lead to neural tube defects suggesting that proper somitic mesoderm development is permissive for neural tube formation.

## Materials and methods

### Xenopus embryos and microinjections

Female adult *X. laevis* frogs were induced to ovulate after injection of human chorionic gonadotropin. Eggs were fertilized *in vitro*, after sacrificing male frogs and acquisition of testes. The embryos were dejellied in 2% cysteine (*pH* 7.8) and subsequently reared in .1 × Marc’s Modified Ringers (MMR). All experiments and experimental protocols were approved by the National Committee for the Protection of Animals Used for Scientific Purposes of the Republic of Cyprus with the license (CY/EXP/PR.L05/2022).

For microinjections, embryos were placed in a solution of 4% Ficoll in .33 × MMR and injected using a glass capillary pulled needle, forceps, a Singer Instruments MK1 micromanipulator and Harvard Apparatus pressure injector at the 4-cell stage according to Nieuwkoop and Faber (Nieuwkoop and Faber, 1994). After injections, embryos were transferred for 1 h in 4% Ficoll in .33 × MMR and then washed and kept in .1 × MMR. Injected embryos were allowed to develop to neurula stages (Nieuwkoop and Faber stage 12-5–13) and imaged live or allowed to develop to the appropriate stage and the dissected or fixed in 1x MEMFA for 1–2 h at room temperature. Capped mRNAs encoding fluorescent protein fusions were *in vitro* transcribed using mMessage machine kits (Ambion). The amount of mRNA per 4 nl of microinjection volume was as follows: membrane-GFP, 80 pg; Utrophin-GFP, 80 pg, FERM-FRNK (FF)-GFP: 500 pg.

### Morpholino oligonucleotides

The Shroom3 (Haigo et al., 2003), Vangl2 (Darken et al., 2002) and Wee2 MO (Leise and Mueller, 2004) morpholinos (MO) were previously described and were ordered from GeneTools. 30 ng of Shroom3 MO, 20 ng of Vangl2 MO, 30 ng Wee2 MO were injected per blastomere.

The amount of MO used was based on what was used in previous studies and typically close to what was shown to elicit maximum downregulation efficiency. Specifically, 20 ng Vangl2 MO was previously used and validated *via* immunofluorescence (Darken et al., 2002; Ossipova et al., 2015). Furthermore, this morpholino at this concentration phenocopies convergent extension defects elicited by dominant negative constructs for PCP proteins (Butler and Wallingford, 2018; Christodoulou and Skourides, 2022). The Shroom3 morpholino has been validated previously and successfully phenocopies the phenotypes induced by a Shroom3 dominant negative construct (Haigo et al., 2003; Lee et al., 2007; Chung et al., 2010). The amount of Shroom3 morpholino previously used for neural plate targeted injections is the same as the amount used in this study (30 ng) (Inoue et al., 2016). The Wee2 morpholino has been validated previously and the amount used in our study a (30 ng) is within the range used previously for efficient Wee2 downregulation (Leise and Mueller, 2004). Shroom3 and Vangl2 MOs were injected at the 2 dorsal blastomeres of 8 cell stage embryos to target the neural plate. Wee2 MO was targeted at the somitic mesoderm by targeted injections laterally of the dorsal marginal zone.

### Immunofluorescence

Immunofluorescence was performed as previously described (Zanardelli et al., 2013). Briefly, embryos were fixed for 2 h at room temperature, permeabilized in PBST (1 × PBS, .5% Triton, 1% dimethyl sulfoxide) and blocked for 1 h in 10% donkey serum. Primary antibodies were incubated overnight at 4°C. We used a primary antibody against integrin MyoD hybridoma supernatant (1:50, D7F2, Hybridoma Bank). Embryos were washed in PBST and incubated for 2 h with secondary antibodies at RT, washed several times and post-fixed in 1XMEMFA. The secondary antibodies used were Alexa fluor 488 (1:500, A21202, Invitrogen), Phalloidin was incubated together with the secondary antibodies, phalloidin 546 (1:500, A22283, Invitrogen).

### Live imaging

Live imaging of neurula stage Xenopus embryos was performed on a ZEISS LSM 710 confocal microscope. The ZEISS ZEN software was used during imaging. Embryos were imaged in a custom chamber made of thick layer of vacuum grease on a microscope slide and sealed with a coverslip. The embryos were imaged in their vitelline membrane, and the height of the custom-made chamber was optimized for each embryo to ensure minimal contact with the coverslip thus achieving immobilization while preventing flattening of the embryos and minimizing external mechanical force application. Embryos were mounted in 0.1 × MMR and kept at room temperature during imaging.

### Image analysis and single cell tracking

**FIGURE 1:**
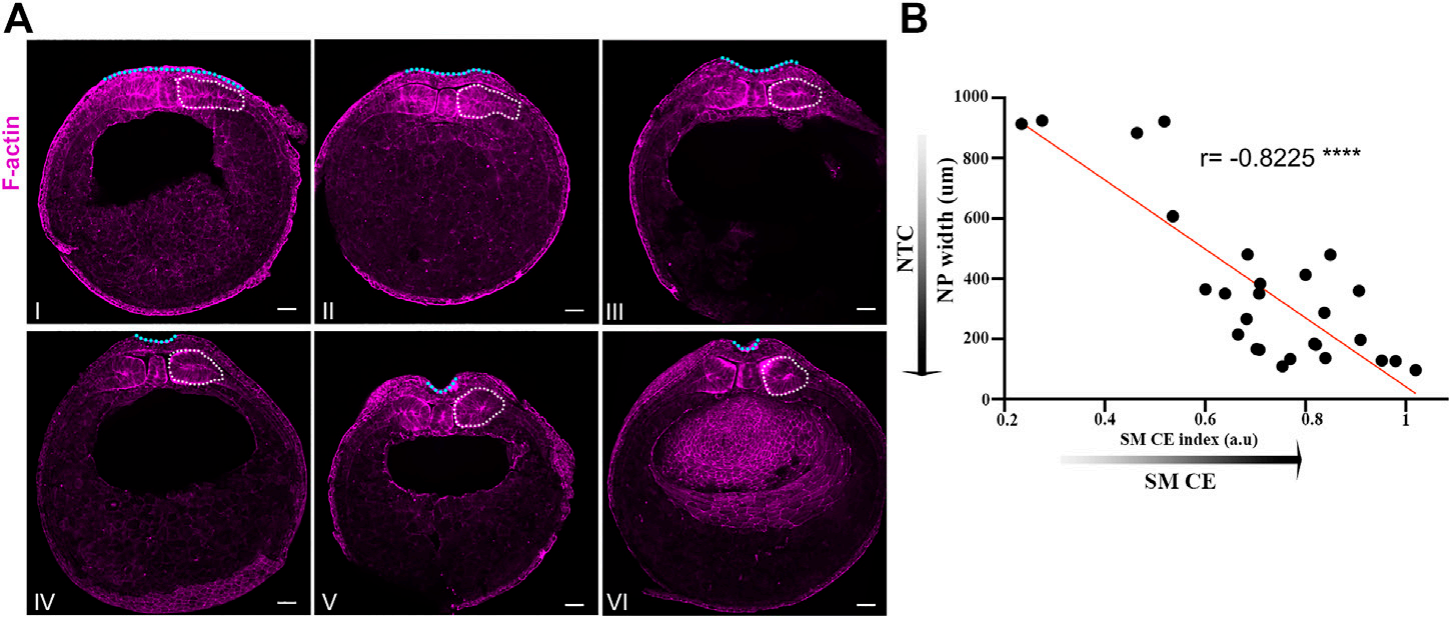
Neural plate and somitic mesoderm morphogenesis are temporally linked during neurulation. **(A)** Transverse cross sections of representative neurula stage Xenopus embryos. Numbering follows neural tube closure progression judged by neural plate mediolateral length. White dotted outline: Somitic mesoderm. Blue dotted line: Neural plate. Scale bars: 100 μm. **(B)** Quantification of neural plate width relative to somitic mesoderm convergent extension index (Somitic mesoderm dorsovetral/mediolateral length). Pearson correlation; ****p < .0001; *n* = 27 embryos. Anti-correlation between neural plate width and somitic mesoderm convergent extension index shows that neural tube closure progression, reduced neural plate width, is highly correlated with progression of somitic mesoderm convergent extension.

**FIGURE 2:**
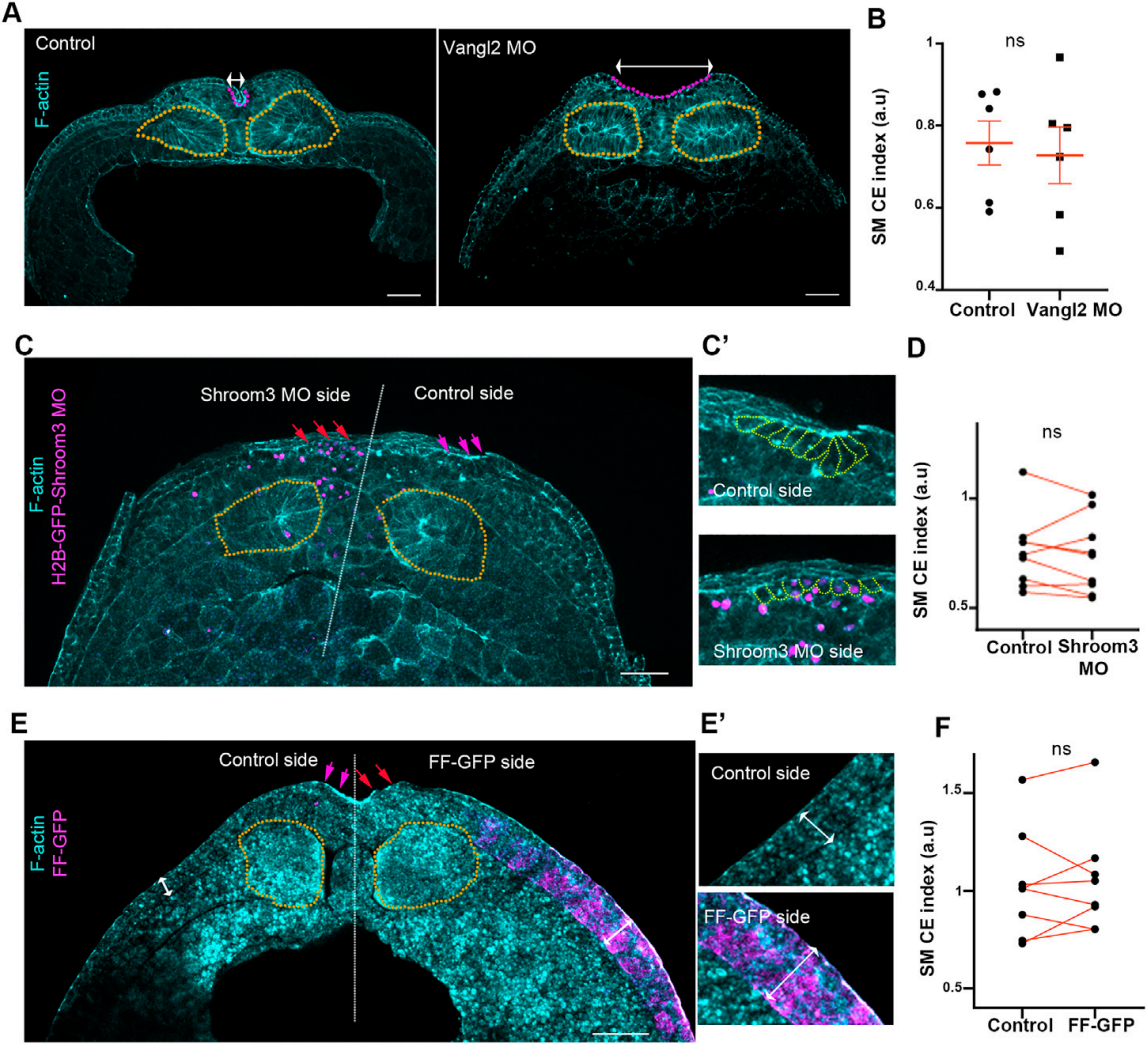
Somitic mesoderm convergent extension occurs independently from neural plate morphogenetic program and surface ectoderm development. **(A)** Representative images of transverse cross sections from control and Vangl2 neural plate morphant stage 16 embryos. Control and Vangl2 morphant embryos are siblings raised side by side. Orange outline: Somitic mesoderm. Purple Outine: neural plate. Double headed arrows: neural plate width. Scale bar: 100 μm **(B)** Quantification of somitic mesoderm convergent extension in control and Vangl2 neural plate morphant embryos. Two-sided unpaired Student’s *t*-test; ns, *p* = .591; mean ± SEM; *n* = 6 embryos. **(C)** Representative images of transverse cross sections from Shroom3 unilateral morphant neural plate stage 16 embryo. Orange outline: Somitic mesoderm. Purple arrows: neural plate hinge point at the control neural plate side. Red arrows: absence of neural plate hinge point at the Shroom3 morphant side. Scale bar: 100 μm **(C’)** Magnified image from neural plate control and Shroom3 morphant side. Orange outlines: neural plate cells. Cells at the control side acquire a wedge shape, characteristic of apical constriction. Cells at the Shroom3 morphant side fail to undergo apical constriction and remain cuboidal. **(D)** Quantification of somitic mesoderm convergent extension in control and Shoom3 morphant neural plate. Two-sided paired Student’s *t*-test; ns, *p* = .516 *n* = 9 embryos **(E)** Representative images of transverse cross sections from a stage 16 embryo expressing FF-GFP at the one side of the surface ectoderm. Orange outline: Somitic mesoderm. Purple arrows: neural plate hinge point at the control neural plate side. Red arrows: absence of neural plate hinge point at the FF-GFP surface ectoderm side. Scale bar: 100 μm **(E’)** Zoomed images of control and FF-GFP surface ectoderm. Double arrowheads: surface ectoderm thickness. Control surface ectoderm is a double layered tissue while the FF-GFP surface ectoderm is composed of multiple cell layers. Phalloidin staining in panel E is weak due to high autofluorescence of yolk platelets within the cells. **(F)** Quantification of somitic mesoderm convergent extension in control and FF-GFP side from embryos displaying unilateral surface ectoderm FF-GFP expression. Two-sided paired Student’s *t*-test; ns, *p* = 0,683; *n* = 8 embryos SM, somitic mesoderm; CE, convergent extension.

**FIGURE 3:**
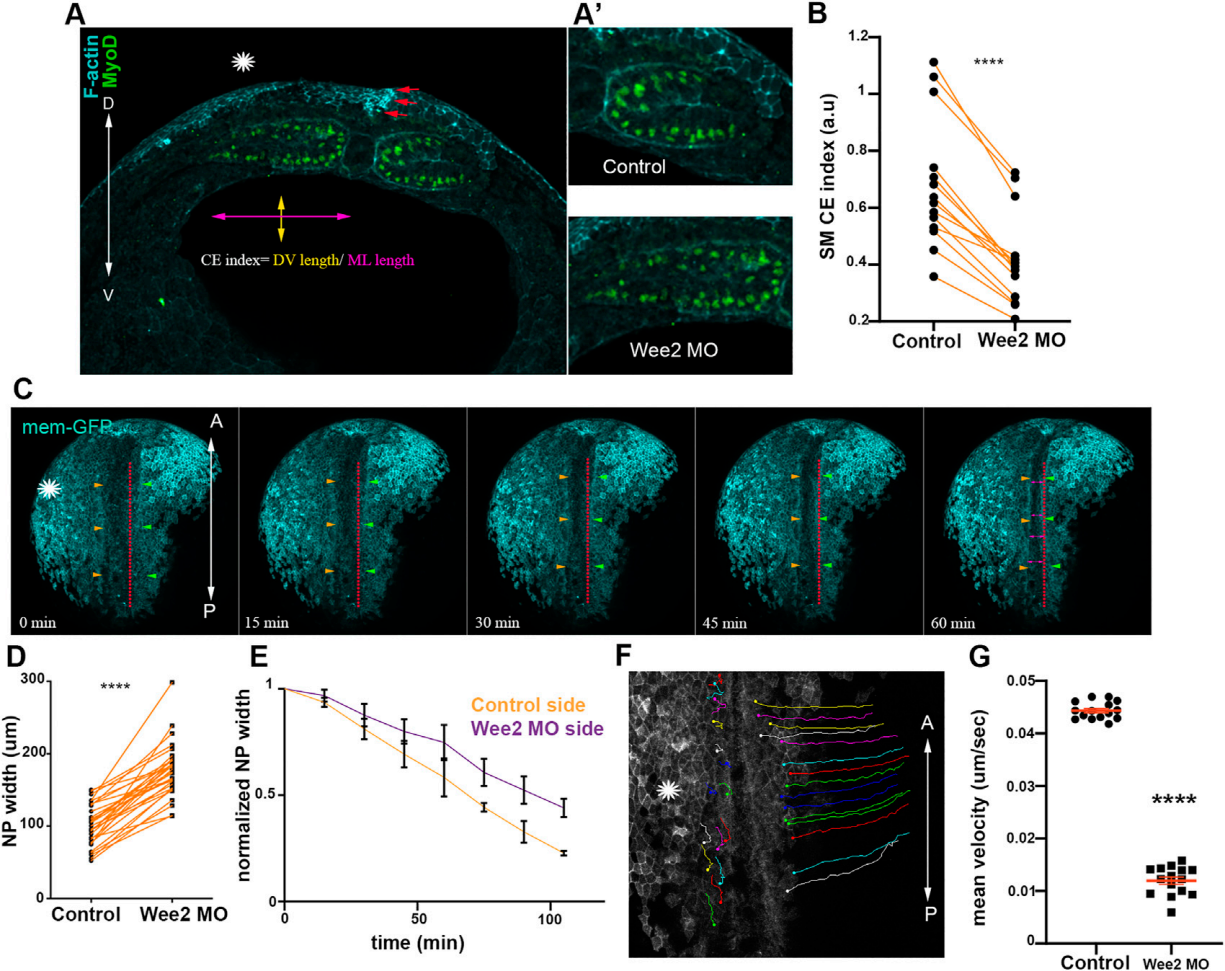
Somitic mesoderm convergent extension in necessary for neural tube closure. **(A)** Transverse cross section a representative Wee2 morphant stage 16 embryo. Yellow double arrowhead: somite dorsoventral (D/V) length. Purple double arrowhead: mediolateral (M/L) somite length. Asterisk indicated the Wee2 morphant side. Red arrows: hinge point at the neural plate side overlying normal somitic mesoderm. **(D)** dorsal. V, ventral. **(A’)** Zoomed images of control and Wee2 morphant somitic mesoderm. Scale bar:100 μm **(B)** Quantification of somitic mesoderm convergent extension index in control Wee2 morphant somitic mesoderm. Two-sided paired Student’s *t*-test; ****p < .0001; *n* = 14 embryos. **(C)** Stills showing the dorsal side of the embryo from a time lapse recording of a neurula embryo with unilateral Wee2 morphant somitic mesoderm. Asterisk: Wee2 morphant side. Red dotted line: neural plate midline. Green arrowheads: Neural plate boundary at the side overlying control somitic mesoderm. Orange arrowheads: Neural plate boundary at the side overlying Wee2 morphant somitic mesoderm. NTC is delayed at the neural plate side overlying Wee2 morphant somitic mesoderm (purple arrowheads). Scale bar: 100 μm **(D)** Quantification of neural plate mediolateral width extension. Neural plate is wider when the underlying somitic mesoderm is targeted with Wee2 morpholino. Two-sided paired Student’s *t*-test; ****p < .0001; *n* = 32 embryos. **(E)** Quantification of neural plate width over time. 3 regions along the anteroposterior axis of a neurula embryo were followed over time. Neural plate width at the side overlying Wee2 morphant somitic mesoderm remains wider as neurulation progresses. **(F)** Dorsal view from a tracked time lapse recording for 1 h time period, showing surface ectoderm boundary cells movement during neural tube closure. Asterisk: Wee2 morphant somitic mesoderm side. **(A)** Anterior; P: Posterior. Scale bar: 100 μm **(G)** Quantification of surface ectoderm boundary cells velocity as a proxy for neural plate hinge point movement. The velocity of surface ectoderm boundary cells is significantly reduced when somitic mesoderm convergent extension is impaired. Two-sided unpaired Student’s *t*-test; ****p < .0001; mean ± SEM; *n* = 15 cells.

**FIGURE 4:**
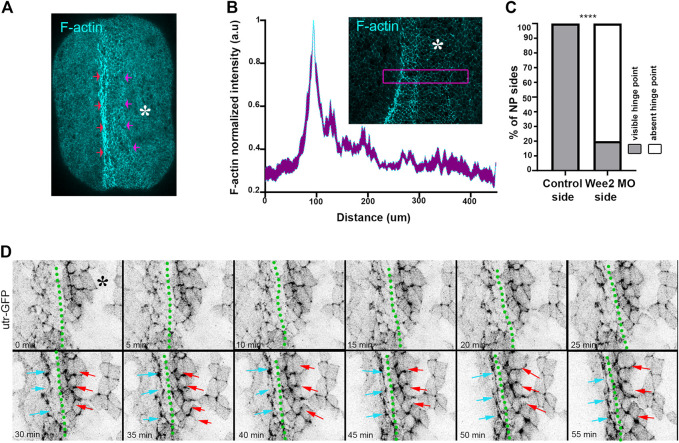
Hinge point formation depend on proper somitic mesoderm morphogenesis. **(A)** Dorsal view of a representative neurula stage 16 embryo stained with Phalloidin (F-actin). Red arrows: F-actin accumulation at the neural plate boundary denotes hinge point formation. Purple arrows: Hinge point formation is impaired at the neural plate side overlying Wee2 morphant somitic medoderm. Asterisk: Side overlying Wee2 morphant somitic mesoderm. Scale bar: 100 μm **(B)** Normalized F-actin intensity profile along the mediolateral neural plate axis. *n* = 10 embryos. Peak intensity indicates the position of the hinge point. Note that F-actin accumulation at the side overlying somitic mesoderm injected with Wee2 morpholino is impaired. Inset: Representative example of neural plate of an embryo with unilateral Wee2 morphant somitic mesoderm. Asterisk: Wee2 morphant side. Purple box was used to plot F-actin intensity profile along the neural plate mediolateral axis. Scale bar: 100 μm **(C)** Quantification of hinge point formation. χ^2^ test; ****p < .0001 *n* = 15 embryos. **(D)** Stills from a time lapse recording of a neurula embryo expressing Utrophin GFP. Green dotted line: midline. Cyan arrows: Neural plate surface ectoderm boundary at the side overlying control somitic mesoderm. Red arrows: Neural plate/surface ectoderm boundary at the neural plate side overlying Wee2 morphant somitic mesoderm. F-actin accumulation characteristic of hinge point formation occurs only at the neural plate/surface ectoderm boundary overlying control somitic mesoderm Scale bar: 50 μm.

**FIGURE 5:**
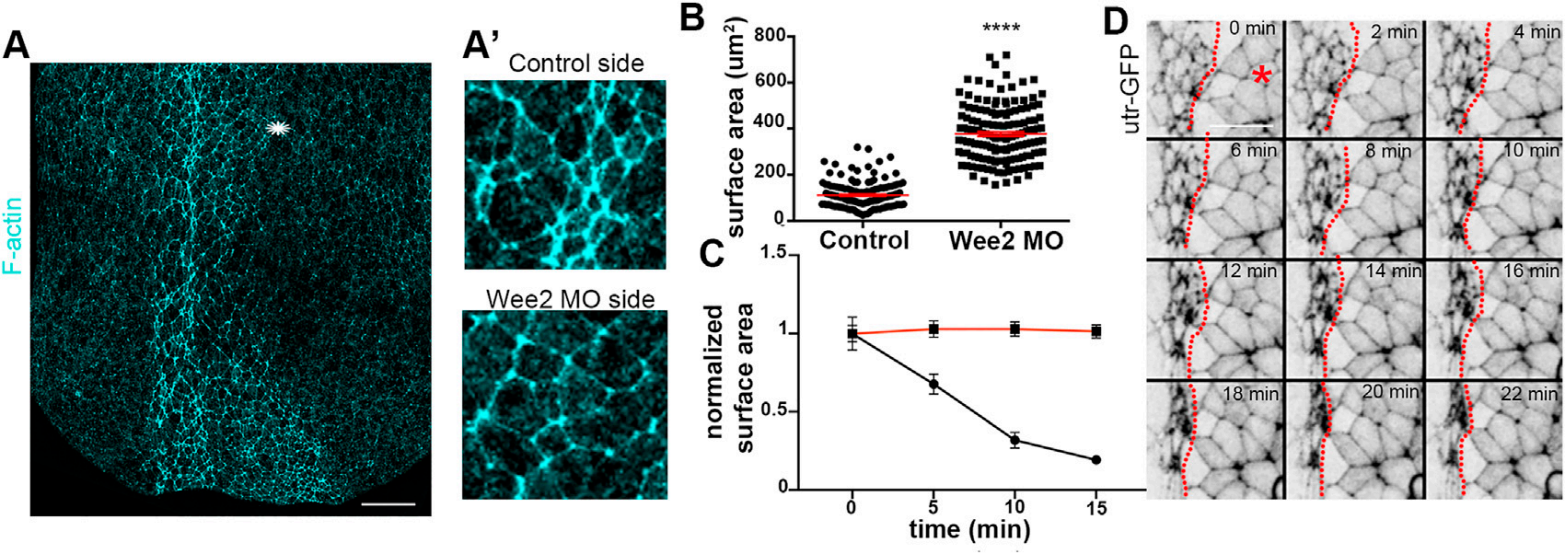
Apical constriction within the neuroepithelium is defective when somitic mesoderm morphogenesis is impaired. **(A)** Maximum intensity projection image of a representative stage 16 embryo. Asterisk: Wee2 somitic mesoderm morphant side. Scale bar: 100 μm **(A’)** Zoomed images of control neuroepithelial cells and neuroepithelial cells overlying Wee2 morphant somitic mesoderm. The apical surface area of neural plate cells overlying the morphant somitic mesoderm side is larger compared to that of control cells. **(B)** Quantification of apical cell surface area. Two-sided unpaired Student’s *t*-test; ****p < .0001; mean ± SEM; *n* = 150 cells. **(C)** Quantification of apical cell surface area over time from neuroepithelial cells in Movie 4 and panel **(D)**; Two-sided unpaired Student’s t-test; ****p < .0001; *n* = 20 cells for each time point. Neuroepithelial cells overlying Wee2 morphant somitic mesoderm fail to undergo apical constriction **(D)** Stills from a time lapse recording of a neurula embryo with unilateral Wee2 morphant somitic mesoderm. Red dotted line: neural plate midline. Asterisk: Wee morphant side. Scale bar: 50 μm.

All image analysis and quantification were carried out using Fiji software (Schindelin et al., 2012). Single cell tracking in Figure 3F was carried out using the manual tracking plugin of Fiji. For somitic mesoderm convergent extension index in Figures 1–3 the dorsoventral length of the presomitic mesoderm was divided by its mediolateral length. Somite boundaries were defined either by cell shape (Somitic mesoderm cells: columnar elongated shape, Lateral plate mesoderm cells: cuboidal shape) for Figures 1, 2 or MyoD expression for Figure 3. The neural plate midline in Figures 3C, 4D, 5D and neural plate boundary in Figure 3C, 4D, were determined by retrograde tracking. The neural plate boundary in Figure 7 was determined based on the elevated F-actin levels in the neural plate compared to those of the surface ectoderm.

### Statistics

GraphPad Prism 8.0 software was used for all statistical analysis performed. The sample size of the experiments carried out was defined based on previous experimental experience. Quantitative data presented, shows the mean ± S.E.M, or the total number of datapoints obtained. The statistical tests carried out on the quantitative data obtained are annotated in each figure legend.

## Results

### Neural tube closure and somitic mesoderm convergent extension display high temporal correlation

We have recently shown that the surface ectoderm is mechanically coupled with the neural plate and its development is permissive for neural tube closure in Xenopus embryos (Christodoulou and Skourides, 2022). Specifically, our recent work revealed that the medial movement of the surface ectoderm during neural tube closure is passive and driven by the remodeling of the neural plate. Additionally, we have shown that increase surface ectoderm tension results in defective neural tube closure. During neural tube closure the neuroepithelium is also mechanically coupled with the underlying somitic mesoderm through the ECM which contains fibronectin, collagen IV, laminin and proteoglycans (Davidson et al., 2004; Mole et al., 2020). Additionally during this stage of development the somitic mesoderm undergoes convergent extension (Wilson et al., 1989) which results in its anteroposterior lengthening, mediolateral thinning and dorsoventral expansion (Leise and Mueller, 2004). At the same time the neural plate lengthens along its anteroposterior axis, thins along the mediolateral axis and bends to form the neural tube (Christodoulou and Skourides, 2015; Christodoulou and Skourides, 2022). We hypothesize that in the event that mesodermal morphogenesis is required for neural plate morphogenesis the evolution of tissue shape changes will be temporally linked. To test this hypothesis, we quantified the width of the neural plate and the degree of somitic mesoderm convergent extension in a mixed population of stage 13 (early neurula) to stage 16 (late neurula) embryos. This analysis revealed that neural tube closure and somitic mesoderm convergent extension are indeed temporally linked (Figures 1A, B). Specifically, neural tube closure progression, evident by the reduction of neural plate width, is accompanied by concomitant dorsoventral expansion and mediolateral thinning of the somitic mesoderm (Figures 1A, B). The tight temporal correlation of the two processes and taking into account the direct interaction of the neuroepithelium with the somitic mesoderm (Davidson et al., 2004), suggest that morphogenetic remodeling in both tissues might result in a reciprocal mechanical communication which will directly impact tissue morphogenesis in either tissue. Such mechanical control of tissue morphogenesis has been described before in other model systems (Butler et al., 2009; Christodoulou et al., 2019; Xiong et al., 2020). Specifically, during Drosophila development, mouse early post-implantation development and chick embryo axis elongation, the behavior of a tissue has a direct impact on its neighboring tissue morphogenesis through mechanical coupling.

### Somitic mesoderm convergent extension is independent of neuroepithelium and SE morphogenesis

Surface ectoderm medial movement at neurula stages is passive and relies on neural plate morphogenesis (Christodoulou and Skourides, 2022). Thus, based on the above data we asked if somitic mesoderm convergent extension is affected by neural plate morphogenesis. To address this possibility, we disrupted neural tube closure by abrogating either convergent extension or apical constriction. To impair neural plate convergent extension, we downregulated a known regulator of convergent extension, Vangl2 (Devenport, 2014), by targeted microinjections of Vangl2 morpholino (Darken et al., 2002). Specifically, to target the neuroepithelium we injected Vangl2 MO at the 2 dorsal/animal blastomeres of 8 cell stage embryos. In order to disrupt apical constriction, we unilaterally downregulated Shroom3 within the neuroepithelium by targeted injections of Shroom3 morpholino (Haigo et al., 2003). As expected, knockdown of both Vangl2 and Shroom3 resulted in impaired convergent extension and apical constriction respectively, as well as defective neural tube closure (Figures 2A, C). Comparison of somitic mesoderm convergent extension in control and Vangl2 morphant embryos, as well as in non-injected and Shroom3 morphant neural plate sides, revealed that somitic mesoderm convergent extension occurs normally both in the absence of neural plate convergent extension and apical constriction (Figures 2A–D). Overall, these data show that despite the direct interaction between the two tissues, somitic mesoderm morphogenesis takes place independently from the neural plate and is not impacted by defects in either neural convergent extension or apical constriction during neural tube closure.

The somitic mesoderm also directly interacts with the surface ectoderm during primary neurulation. During mouse embryogenesis, after completion of neural tube closure paraxial mesoderm morphogenesis depends on the presence of the surface ectoderm (Correia and Conlon, 2000). Therefore, we went on to examine if surface ectoderm development affects somitic mesoderm convergent extension during neural tube closure stages. For this, we used a previously characterized dominant negative construct for FAK, FERM-FRNK (FF), which has been shown to lead to thickening and stiffening of the ectoderm epithelium during gastrula and neurula stages (Petridou et al., 2013; Christodoulou and Skourides, 2022). As expected, unilateral targeted injections of FF-GFP led to thickening of the surface ectoderm. This was accompanied by defects in neural tube closure (Figure 2E), as previously described (Christodoulou and Skourides, 2022). In contrast to the effect on neural plate, SE thickening did not affect somitic mesoderm convergent extension during neural tube closure stages (Figure 2F). These data indicate that somitic mesoderm convergent extension occurs independently of surface ectoderm expansion.

Overall, the above data demonstrate that somitic mesoderm convergent extension is an active process and occurs independent from both neural plate and surface ectoderm morphogenesis. Importantly, these data are in agreement with the fact that somitic mesoderm explants can undergo convergent extension in the absence of the neuroepithelium and the surface ectoderm (Wilson et al., 1989). Our data further show that in the presence of neural plate and surface ectoderm defects somitic mesoderm morphogenesis is unaffected. The latter is in agreement with data demonstrating that the paraxial mesoderm is twice as stiff as the neuroepithelium at neurula stages (Zhou et al., 2009). This implies that mechanical resistive forces generated by a deformed neuroepithelium, or surface ectoderm are not able to impede morphogenesis of the stiffer somitic mesoderm which can generate forces that can overcome softer tissue resistance.

### Somitic mesoderm convergent extension is necessary for hinge point formation neural tube closure

Neither neural plate nor surface ectoderm defects can appreciably impact somitic mesoderm morphogenesis likely due to the higher stiffness and resulting elevated force generation capacity of the somitic mesoderm compared to that of adjacent tissues. This would presumably allow somitic mesoderm morphogenesis to elicit passive deformation of adjacent tissues moderating potential defects from arising in the stiffer tissue when surrounding softer tissues that are not properly formed. Based on this interpretation, it would be expected that defects in the remodeling of the somitic mesoderm would mechanically impact the morphogenesis of the neuroepithelium. We went on to examine the impact of somitic mesoderm morphogenesis on neural tube closure. To accomplish this, we decided to specifically disrupt somitic mesoderm convergent extension during Xenopus neurulation. The tyrosine kinase Wee2 has been previously shown to display tissue restricted expression within the paraxial mesoderm (Leise and Mueller, 2004). Importantly, Wee2 is responsible for the transient cell cycle arrest within this tissue, which is necessary for somitic mesoderm convergent extension (Leise and Mueller, 2004). Therefore, Wee2 is an ideal target to specifically disrupt somitic mesoderm convergent extension during neurulation. We proceeded with targeted unilateral microinjection of a previously characterized morpholino against Wee2 (Leise and Mueller, 2004), targeting the paraxial mesoderm (Supplementary Figures S1A, B). Upon Wee2 downregulation, Wee2 morphant somitic mesoderm failed to thin along the mediolateral axis and expand along the dorsoventral embryo axis (Figures 3A, B), showing that Wee2 downregulation results in impaired somitic mesoderm convergent extension, as previously described (Leise and Mueller, 2004).

Having established an effective approach to specifically to disrupt somitic mesoderm convergent extension, we went on to examine the impact of defective somitic mesoderm convergent extension on neural tube closure. We performed live imaging of Wee2 morphant embryos and analyzed fixed morphant embryos. These experiments revealed that unilateral defects in somitic mesoderm convergent extension resulted in defective neural tube closure (Figures 3C–E; Supplementary Figure S1C; Supplementary Movie S1). Specifically, analysis of neural plate width in fixed embryos revealed that the NP was wider at the side injected with Wee2 MO (Figure 3D; Supplementary Figure S1C). Furthermore, time lapse recording revealed slower movement of neural folds towards the midline of the neural plate resulted in wider NP at the Wee2 morphant side (Figures 3E–G; Supplementary Movie S2). Overall, these data show that somitic mesoderm convergent extension is necessary for neural tube closure. Our data are in agreement with recent studies showing that paraxial mesoderm morphogenesis has a positive impact on neural tube closure during mouse embryogenesis (de Goederen et al., 2022; Li et al., 2022; Nychyk et al., 2022). However, what aspects of neural plate morphogenesis are affected by defects in somitic mesoderm development is not known.

Hinge points form at the lateral edges of the neural plate and are visible as areas with increased actomyosin accumulation (Haigo et al., 2003; Christodoulou and Skourides, 2015). Examination of paraxial mesoderm Wee2 morphant neurula stage embryos revealed that F-actin accumulation at the edge of the neural plate overlying the Wee2 morphant somitic mesoderm was abolished (Figures 4A–C). Based on these observations we conclude that hinge point formation is defective when somitic mesoderm morphogenesis is impaired. F-actin cable establishment and maintenance are both sensitive to tension forces and actin filaments have been suggested to directly sense mechanical force (Sun et al., 2020; Winkelman et al., 2020). Hingepoints, are cable like structures which are under constant mechanical stimulation, since are located at the boundary of the actively remodeling neural plate. We decided to examine if somitic mesoderm convergent extension is needed for the initial formation or is necessary for the subsequent maintenance of the hinge point. To accomplish this, we performed live imaging using Wee2 morphant embryos, injected with Utrophin-GFP, an F-actin marker compatible with live imaging (Christodoulou and Skourides, 2015; Christodoulou and Skourides, 2022). Time lapse-recording revealed that the hinge point, indicated by the reduction of cell surface area and subsequent accumulation of medioapical F-actin, fails to form at the neural plate side overlying Wee2 morphant somitic mesoderm (Figure 4D; Supplementary Movie S3). This shows that somitic mesoderm morphogenesis is necessary for the initial formation of hinge points during neural tube closure.

### Somitic mesoderm convergent extension is a prerequisite for neuroepithelial cell apical constriction

Hinge points form upon dramatic change of the shape of neural plate lateral boundary cells (Haigo et al., 2003; Christodoulou and Skourides, 2015). This cell shape change of boundary cells is a consequence of apical constriction (Christodoulou and Skourides, 2015). The defects in hinge point formation when somitic mesoderm convergent extension is impaired, suggest that apical constriction within the neuroepithelium requires proper somitic mesoderm shaping. The hallmark of apical constriction is the reduction of apical cell surface area (Sawyer et al., 2010; Martin and Goldstein, 2014). Quantification of the apical surface area of neuroepithelial cells from control or morphant somitic mesoderm in fixed embryos revealed that the apical surface of neuroepithelial cells overlying Wee2 morphant somitic mesoderm was larger (Figures 5A-A’, B). Additionally, time lapse recordings of embryos expressing Utr-GFP and have unilateral Wee2 morphant somitic mesoderm revealed that neuroepithelial cells at the morphant side fail to reduce their apical surface area (Figures 5C, D; Supplementary Movie S4). These data show, as expected based on the lack of hinge point formation, that apical constriction within the neuroepithelium is defective when somitic mesoderm convergent extension is impaired. Importantly examination of neighbor exchanges, a cell behavior necessary for neural plate convergent extension (Blankenship et al., 2006; Butler and Wallingford, 2018; Christodoulou and Skourides, 2022), revealed that this behavior is unaffected in Wee2 morphants (Supplementary Figure S2; Supplementary Movie S5), suggesting that somitic mesoderm convergent extension specifically impacts neuroepithelial cell apical constriction. The latter is partially supported by data showing that deep neural tissue explants autonomously undergo convergent extension (Elul et al., 1997).

**FIGURE 6:**
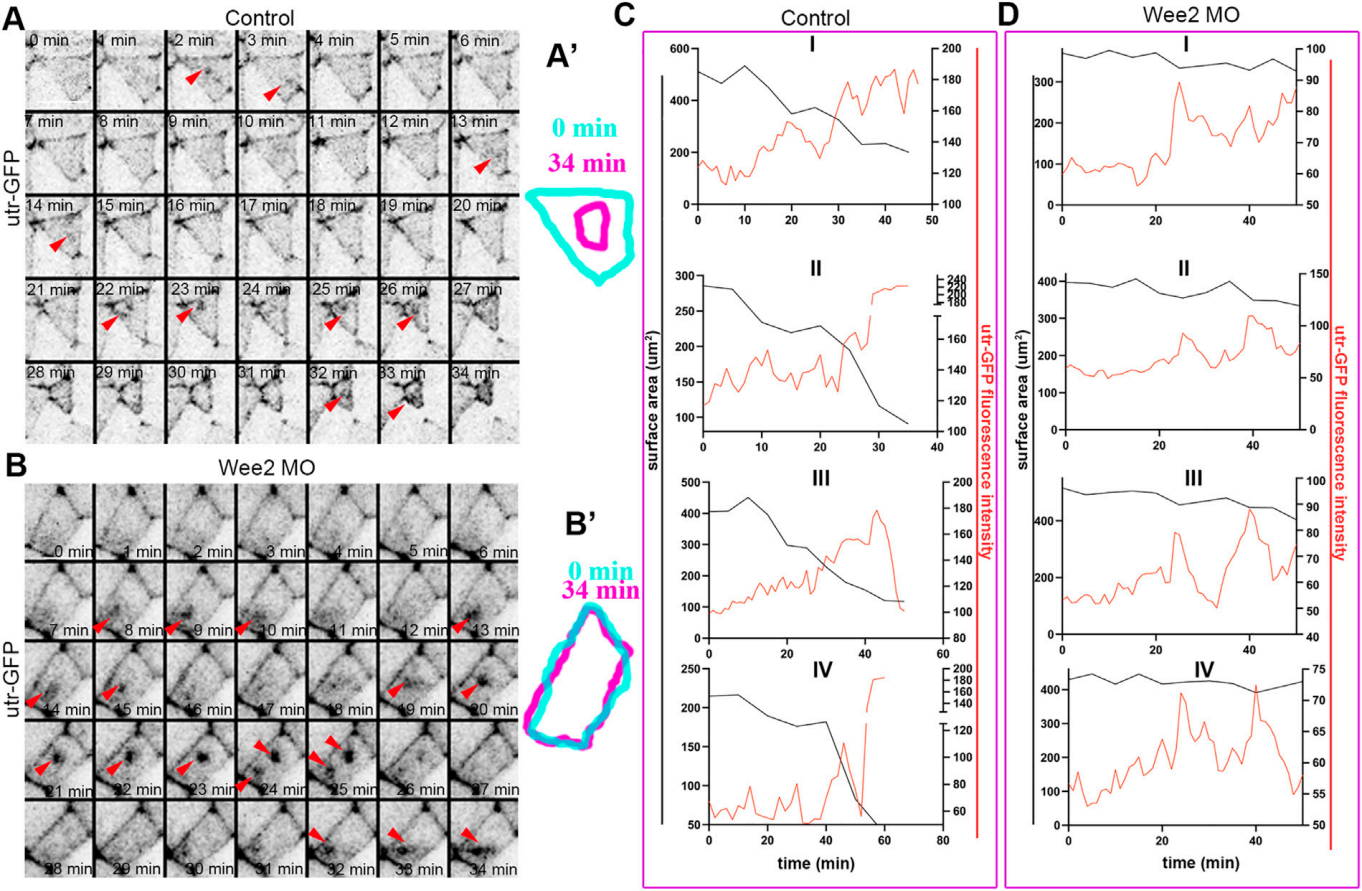
Somitic mesoderm morphogenesis affects the ratchetting of contraction pulses during apical constriction. **(A)** Stills from a time lapse recording of a control neuroepithelial cell during apical constriction. Red arrowheads: pulsed medioapical actin accumulation. Scale bar: 20 μm. **(A’)** Outline of the apical cell surface area at 0 and 34 min showing reduction of apical cell surface area. **(B)** Stills from a time lapse recording of a neuroepithelial cell overlying Wee2 morphant somitic mesoderm during apical constriction. Red arrowheads: pulsed medioapical actin accumulation. Scale bar: 20 μm. **(B’)** Outline of the apical cell surface area at 0min and 34 min showing defective reduction of apical cell surface area. **(C,D)** Quantification of medioapical actin intensity and apical cell surface area over time. **(C)** 4 control neuroepithelial cells. **(D)** 4 neuroepithelial cells overlying morphant somitic mesoderm. Medioapical actin accumulation (red line) is followed by reduction of the cell surface area (black line) in control cells. The cell surface area is not reduced upon apical F-actin accumulation in neuroepithelial cells overlying morphant somitic mesoderm.

Apical constriction during neural tube closure is driven by calcium mediated actomyosin contraction pulses (Christodoulou and Skourides, 2015). The stepwise reduction of the apical cell surface is maintained by a ratchet mechanism which prevents cell relaxation between each contraction step (Martin et al., 2009; Krueger et al., 2020). We and others have shown that the contraction and stabilization steps during apical constriction have distinct regulators (Martin et al., 2009; Christodoulou and Skourides, 2015). To examine which step of apical constriction is affected by defects in somitic mesoderm morphogenesis we analyzed time lapse recordings of embryos expressing the F-actin marker Utrophin-GFP. Our analysis revealed pulsed accumulation of medioapical F-actin in neuroepithelial cells overlying control somitic mesoderm. Similarly, the pulsed accumulation of medioapical actin was present in neuroepithelial cells overlying Wee2-morphant somitic mesoderm (Figures 6A,A’,B,B’; Supplementary Figures S3A–C; Supplementary Movie S6, S7). As shown medioapical actin accumulation is followed by a reduction of the apical cell surface area in neuroepithelial cells overlying normal somitic mesoderm. In contrast, accumulation of medioapical actin is not followed by efficient reduction of the apical surface in cells overlying morphant somitic mesoderm (Figures 6C,D; Supplementary Figure S3D; Supplementary Movie S6, S7). This phenotype is reminiscent of defects in apical ratcheting during apical constriction (Krueger et al., 2020). These findings suggest that while the genetic program necessary for the initiation of contraction pulses during neuroepithelial cell apical constriction is unaffected, tensile forces exerted on the neuroepithelium by the mispositioned underlying somitic mesoderm affect the stabilization of the actomyosin machinery necessary for ratcheted apical constriction, possibly through a mechanosensitive signaling pathway. Importantly, the molecular regulators of apical constriction have been shown to be mechanosensitive (Pouille et al., 2009). Thus, our data suggest that increased resistive forces stemming from defective somitic mesoderm morphogenesis impact on mechanosensitive molecules regulating actomyosin contractility during apical constriction.

### Additive contribution of abnormalities in neuroepithelium and somitic mesoderm morphogenesis leads to defective neural tube closure

**FIGURE 7:**
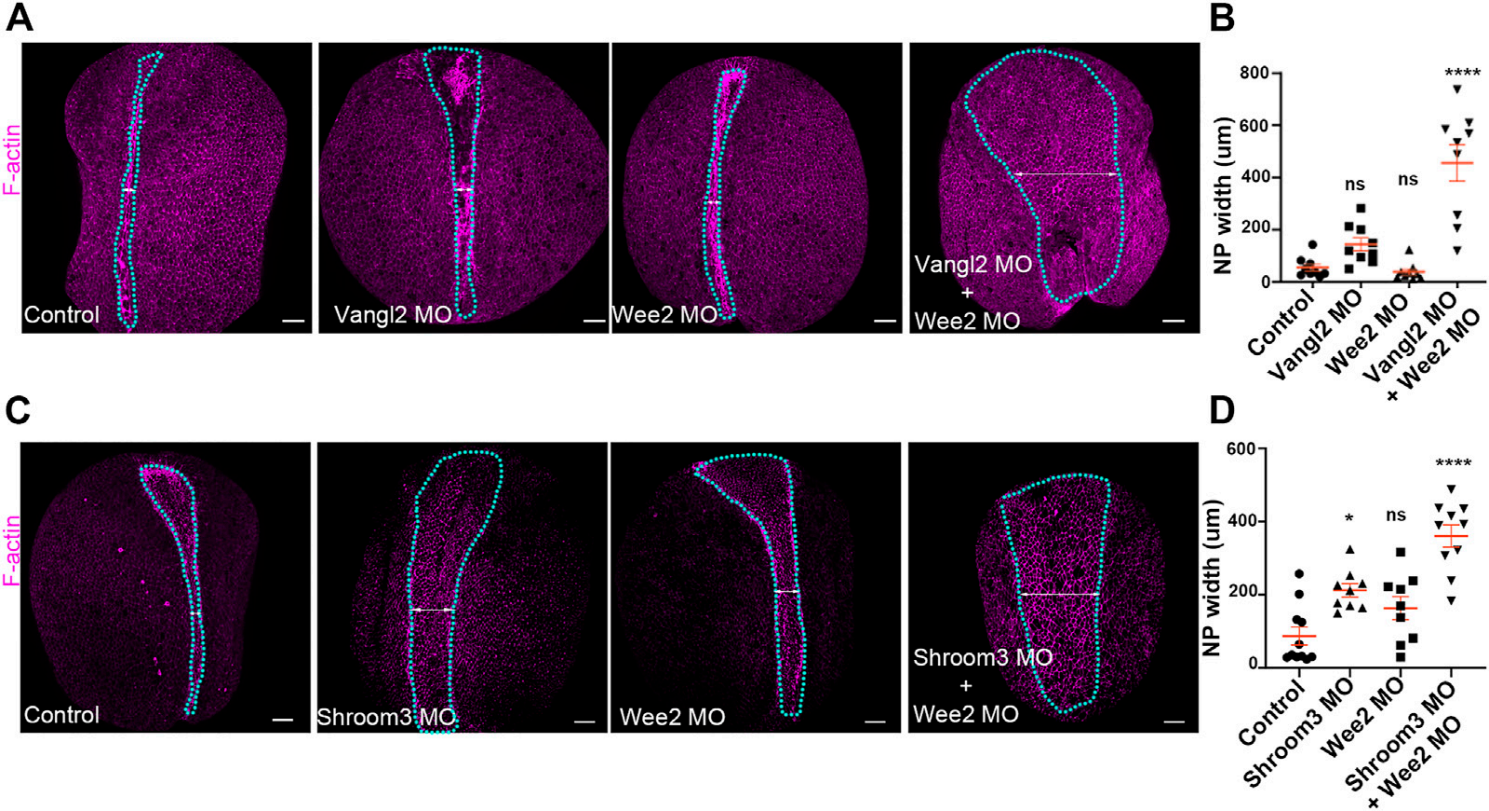
Defects in neural plate and somitic mesoderm morphogenesis have additive impact on neural tube closure. **(A)** Dorsal views of representative Control, Vangl2 morphant, Wee2 morphant and Vangl2/Wee2 double morphant embryos. Blue outline: neural plate. Double headed arrow: neural plate width. Scale bars: 100 μm. Control and morphant embryos are siblings raised side by side **(B)** Quantification of neural plate width in Control, Vangl2 morphant, Wee2 and Vangl2/Wee2 double morphant embryos. Neural tube closure is severely affected in Vangl2/Wee2 double morphant embryos. One-way Anova; ns: no significant; ****p < .0001; mean ± SEM *n* = 9 embryos. **(C)** Dorsal views of representative Control, Shroom3 morphant, Wee2 morphant and Shroom3/Wee2 double morphant embryos. Blue outline: neural plate. Double headed arrow: neural plate width. Scale bars: 100 μm. Control and morphant embryos are siblings raised side by side **(D)** Quantification of neural plate width in Control, Shroom3 morphant, Wee2 morphant and Shroom3/Wee2 double morphant embryos. Neural tube closure is severely affected in Shroom3/Wee2 double morphant embryos. One-way Anova; ns: no significant; *p = .106: ****p < .0001; mean ± SEM *n* = 11 control, 9 Shroom3 and Wee2 morphant and 10 Wee2/Shroom3 double morphant embryos.

Collectively, our data indicate that somitic mesoderm morphogenesis is necessary for neural tube closure. They also show that defects in somitic mesoderm convergent extension directly impact intrinsic neural plate morphogenesis, specifically apical constriction. Given the above, we postulated that mild defects in neural plate and somitic mesoderm morphogenesis will have an additive contribution on neural plate morphogenesis. To examine this possibility, we performed targeted injections using sub-optimal (half the amount of morpholino inducing a phenotype) amounts of Vangl2, Shroom3 and Wee2 morpholino. Specifically, we initially targeted the paraxial mesoderm with Wee2 morpholino, by injecting laterally of the dorsal marginal zone of 4 cell stage embryos. Subsequently half of those embryos at the 8-cell stage were injected with Vangl2 or Shroom3 morpholino at the 2 dorsal animal blastomeres at the. Uninjected 8 cell stage embryos were injected at the two dorsal blastomeres with suboptimal amount of only Shroom3 or only Vangl2 morpholino to obtain Vangl2 and Shroom3 single morphant embryos. Subsequently we assessed neural tube closure progression in control embryos, Wee2 single morphants, and Wee2/Vangl2 or Shroom3 double morphant embryos by quantifying neural plate width. This analysis revealed mild neural tube defects in Wee2, Vangl2 and Shroom3 single morphant embryos (Figures 7A–D). In contrast, in Wee2/Vangl2 and Wee2/Shroom3 double morphant embryos neural tube closure was significantly affected (Figures 7A–D). These data suggest that severe neural tube defects might emerge when a combination of mild defects in neural tube closure and somitic mesoderm morphogenesis co-exist. This might be the case, in severe neural tube defects emerging in otherwise normal LP/+ heterozygote mouse embryos when treated with chlorate (Nychyk et al., 2022). Chlorate is a potent inhibitor of CAG sulfation. Inhibition of CAG sulfation in LP/+ heterozygote mouse embryos, in which PCP signaling is partially lost, results in neural tube defects, which have been attributed to abnormal somite morphology.

## Discussion

During neurulation both the neural plate and the somitic mesoderm undergo dramatic morphogenetic remodeling. Additionally, during vertebrate neurulation these tissues change their relative position. Specifically, while at the beginning of neurulation the somitic mesoderm is found subjacent of the neural plate, by the end of neurulation, the somitic mesoderm flanks the neural tube. These tissues are mechanically coupled through their attachment to a common ECM rich in fibronectin, collagen IV and laminin and proteoglycans (Davidson et al., 2004; Mole et al., 2020). In this study we examine the reciprocal mechanical impact of neural tissue morphogenesis and somitic mesoderm morphogenesis. Initially, our data reveal that neural plate and somitic mesoderm development are temporally linked during neurulation. Next, we show that somitic mesoderm morphogenesis can occur independently from both neural plate intrinsic morphogenesis and surface ectoderm behaviour. Subsequently, our findings reveal that somitic mesoderm morphogenesis is necessary for neural tube closure. Specifically, we show that defects in somitic mesoderm convergent extension result in impaired hinge point formation due to defective apical constriction within the neural plate. Last, we show that somitic mesoderm and neural plate morphogenesis synergize for neural tube formation and defects in somitic mesoderm and neural plate morphogenesis have additive impact on neural tube closure. Our work adds to recently published work (de Goederen et al., 2022; Li et al., 2022; Nychyk et al., 2022) on the role of paraxial mesoderm during central nervous system morphogenesis since we show for that defective somitic mesoderm morphogenesis is necessary for apical constriction within the neural plate.

The mechanical force generators that drive neural tube closure are both intrinsic (forces generated within the neural plate) and extrinsic (originating in tissues displaying mechanical linkage with the neural plate). Although intrinsic force generation is well documented, neural tube closure is a complex biomechanical event in which external influences are still poorly understood. Here, we show that somitic mesoderm morphogenesis occurs independently from the behavior of both the neuroepithelium and the surface ectoderm. This is in agreement with classic explant experiments, showing that dorsal paraxial mesoderm explants undergo convergent extension in the absence of both overlying tissues, the neuroepithelium and the surface ectoderm (Wilson et al., 1989). Furthermore, the relative stiffness of the three tissues can explain the independent behavior of the somitic mesoderm. Tissue stiffness in a given tissue will be affected by the underlying tissues. Thus, neural plate stiffness will be heavily influenced by the mechanical properties of the underlying notochord, somitic mesoderm and neural plate. Previous studies measured the tissue stiffness of individual tissues from Xenopus neurula stage embryos using tissue explants (Wiebe and Brodland, 2005; Zhou et al., 2009). These studies revealed that surface ectoderm is less stiff when compared with the neural plate (Wiebe and Brodland, 2005). Importantly, during neurula stages somitic mesoderm stiffness is higher compared with the stiffness of the neural plate (Zhou et al., 2009). Thus, while neural plate medial movements are driving the movement of the less stiff surface ectoderm (Christodoulou and Skourides, 2022), neural plate behavior cannot mechanically impact the morphogenesis of the stiffer somitic mesoderm.

In contrast with the independence of somitic mesoderm convergent extension from neural plate morphogenesis, neural tube closure depends on the somitic mesoderm. Analysis of fixed samples and time lapse recordings revealed that somitic mesoderm convergent extension is essential for apical constriction and hinge point formation. Specifically, defective somitic mesoderm morphogenesis affects ratcheted contraction pulses while the pulsed accumulation of medio apical actin is unaffected. It is known that actomyosin dynamics, which regulate apical constriction (Martin et al., 2009), are mechanosensitive (Fernandez-Gonzalez et al., 2009; Pouille et al., 2009). Thus, our data suggest that tensile forces exerted on the neuroepithelium by the mispositioned somitic mesoderm affect mechanosensitive regulators of apical constriction. Alternatively, the resistive forces established by defective mesoderm morphogenesis may simply be beyond the ability of apical constriction to overcome. We currently cannot distinguish between the two possibilities however the establishment of mediopacial actin suggests that the signaling and cellular machinery responsible for apical constriction are functioning normally and increased tissue tension leads to surface reduction failure. Future studies exploring changes of the tension landscape within the neural plate in response to paraxial mesoderm defects should be able to directly address these possibilities.

While our findings clearly show that somitic mesoderm morphogenesis is necessary for neural tube closure, we cannot explicitly state if somitic mesoderm convergent extension generates active forces for the folding of the neural plate, or if somitic mesoderm remodeling through convergent extension is permissive for neural tube closure. However, the knowledge acquired by this study in combination with classical work on Xenopus embryo tissue explants, suggest that somitic mesoderm morphogenesis does not actively contribute to neural tube morphogenesis but is permissive for neural tube closure. Specifically, when the neural plate is explanted without the underlying mesoderm, neuroepithelial cells efficiently undergo apical constriction acquiring a wedge shape. Furthermore, in these explants the hinge points are formed and the tissue bends (Poznanski et al., 1997). Therefore, the fact that neuroepithelial cells in these neural explants which lack somitic mesoderm can undergo apical constriction and form hinge points suggests that somitic mesoderm morphogenesis is permissive for neuroepithelial cell apical constriction and does not actively contribute to the folding of the neuroepithelium. However, it is possible that somitic mesoderm morphogenesis actively contributes to the last phase of neural tube formation since only neural plate explants with somitic mesoderm present undergo successfully radial intercalation and form a neural tube (Poznanski et al., 1997). Future work should focus on the delineation of somitic mesoderm contribution during different stages of neural tube closure.

Here, we have shown for the first time that defects in neural plate and somitic mesoderm morphogenesis have additive effect on neural tube closure. Mild defects in neural plate and somitic mesoderm morphogenesis can result in severe neural tube defects. Knock-out of more than 200 genes have been shown to result in neural tube defects in mouse embryos (Juriloff and Harris, 2000; Harris and Juriloff, 2007; Harris and Juriloff, 2010). However, a lot of these genes are ubiquitously expressed and can affect both neural plate and somitic mesoderm morphogenesis at the same time. Therefore, to delineate the role of these genes in neural plate and somitic mesoderm morphogenesis tissue specific knockout mice will have to be generated in the future. With this approach we will be able to uncover if neural tube defects observed upon gene depletion is dependent on the role of a given gene in the neural plate morphogenesis, somitic mesoderm morphogenesis or if the function of a gene product is necessary for proper morphogenesis of both tissues.

Our previous work as well as work from others has highlighted the importance of proper surface ectoderm development for neural tube closure (Pyrgaki et al., 2011; Nikolopoulou et al., 2019; Christodoulou and Skourides, 2022). This work and recently published work highlight the contribution of somitic mesoderm morphogenesis during neural tube formation (de Goederen et al., 2022; Li et al., 2022; Nychyk et al., 2022). Collectively, the presented data show that defects in mechanically coupled tissue morphogenesis result in neural tube defects. We propose that neural tube defects can be categorized in 1) neural tube defects emerging from defects of neural plate morphogenesis; and 2) neural tube defects emerging from defects in mechanically coupled tissues. The appearance of neural tube defects in human births has been significantly reduced due to folic acid supplementation (Wilde et al., 2014; Crider et al., 2022). However, is it well known that at least one third of neural tube defects are not responsive to folate (Greene and Copp, 2014). It is possible that the distinct categories of neural tube defects will respond differently to folate supplementation. Thus, it will be important to study neural tube defects emerging from defects in mechanically coupled tissues and assess the responsiveness of such neural tube defects to folate supplementation in future studies.

## Data Availability

The original contributions presented in the study are included in the article/Supplementary Material, further inquiries can be directed to the corresponding authors.
