# Supplementary material for: Somitic mesoderm morphogenesis is necessary for neural tube closure during Xenopus development
Source: Front Cell Dev Biol. 2023 Jan 9;10:1091629. doi: 10.3389/fcell.2022.1091629 (PMC9868421; doi:10.3389/fcell.2022.1091629)
Supplement: Supplementary file 3 [file DataSheet1.PDF]

Supplementary Material

Supplementary Figures

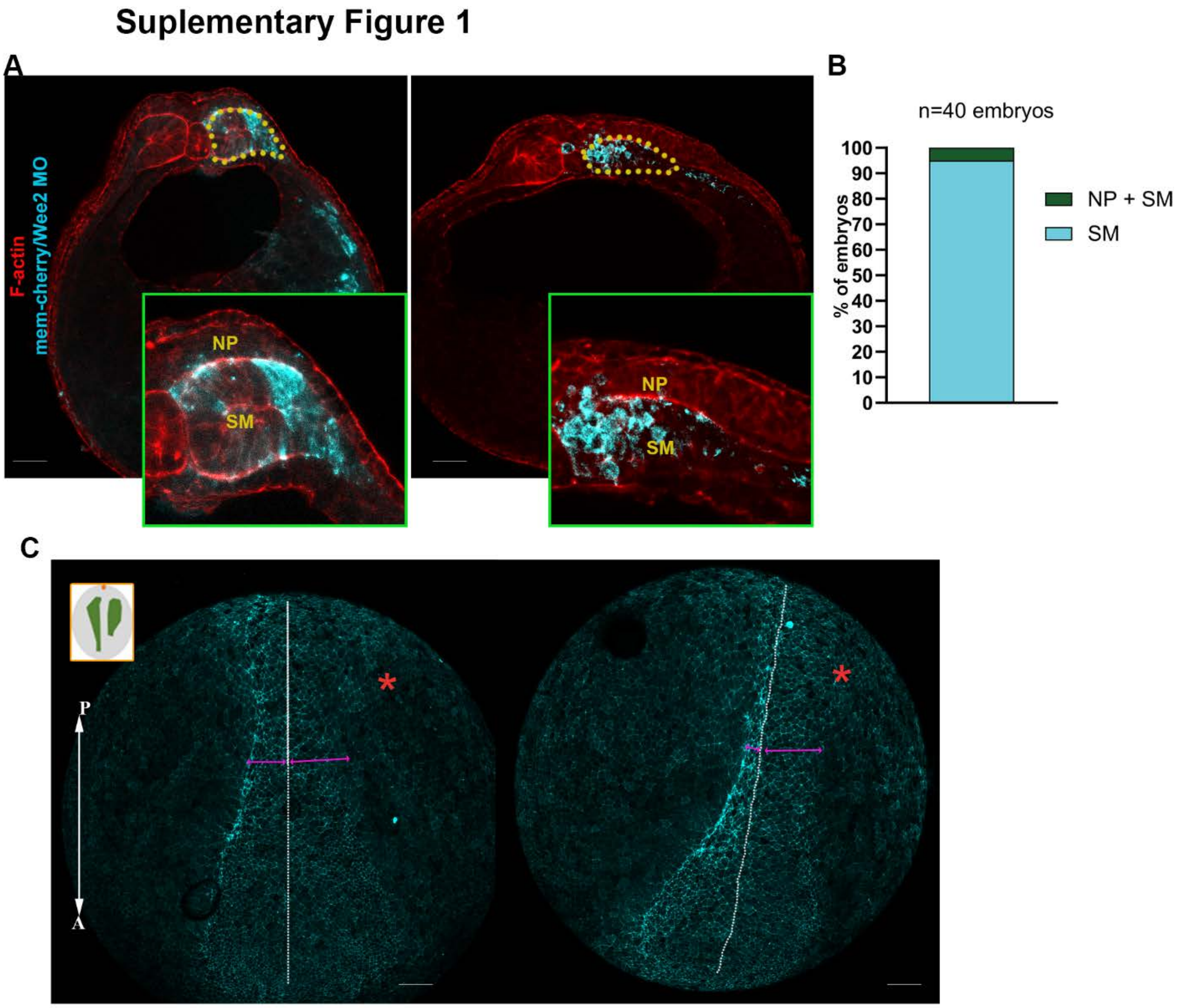

Supplementary Figure 1. Somitic mesoderm targeted Wee2 knockdown results in defective neural tube closure. A) Representative cross sections of Wee2 morphant embryos showing somitic mesoderm specific targeting as shown by the localization of the linage tracer mem-cherry. Orange outline: Insets showing magnified images of somitic mesoderm (SM) and overlying neural plate (NP)**B)** Quantification of lineage tracer distribution after unilateral targeted microinjection of Wee2 morpholino. NP: neural plate, SM: somitic mesoderm. **C)**Maximum intensity profile images of representative embryos with unilateral Wee2 morphant somitic mesoderm. Asterisk: morphant side. Scale bars: 100 um.

Supplementary Figure 2

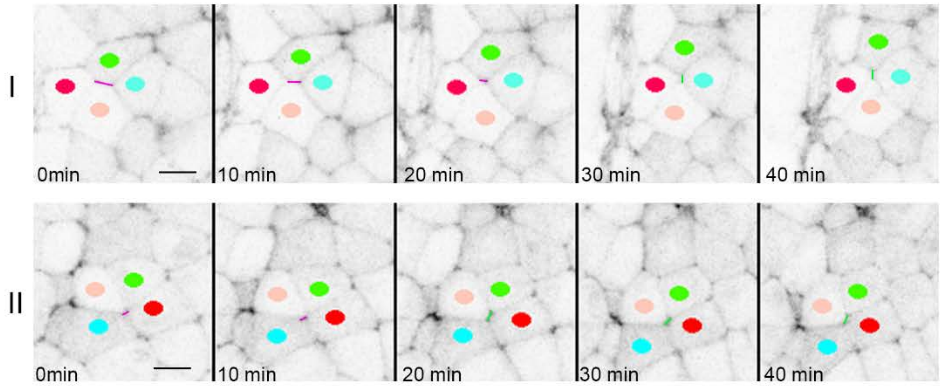

Supplementary Figure 2: Neighbor exchanges in neural plate overlying Wee2 morphant somitic mesoderm. Example of neighbor exchanges in the neuroepithelium overlying Wee2 morphant somitic mesoderm. Purple line: Shrinking junction. Green Line: Elongating junction. Scale bar: 100 um.

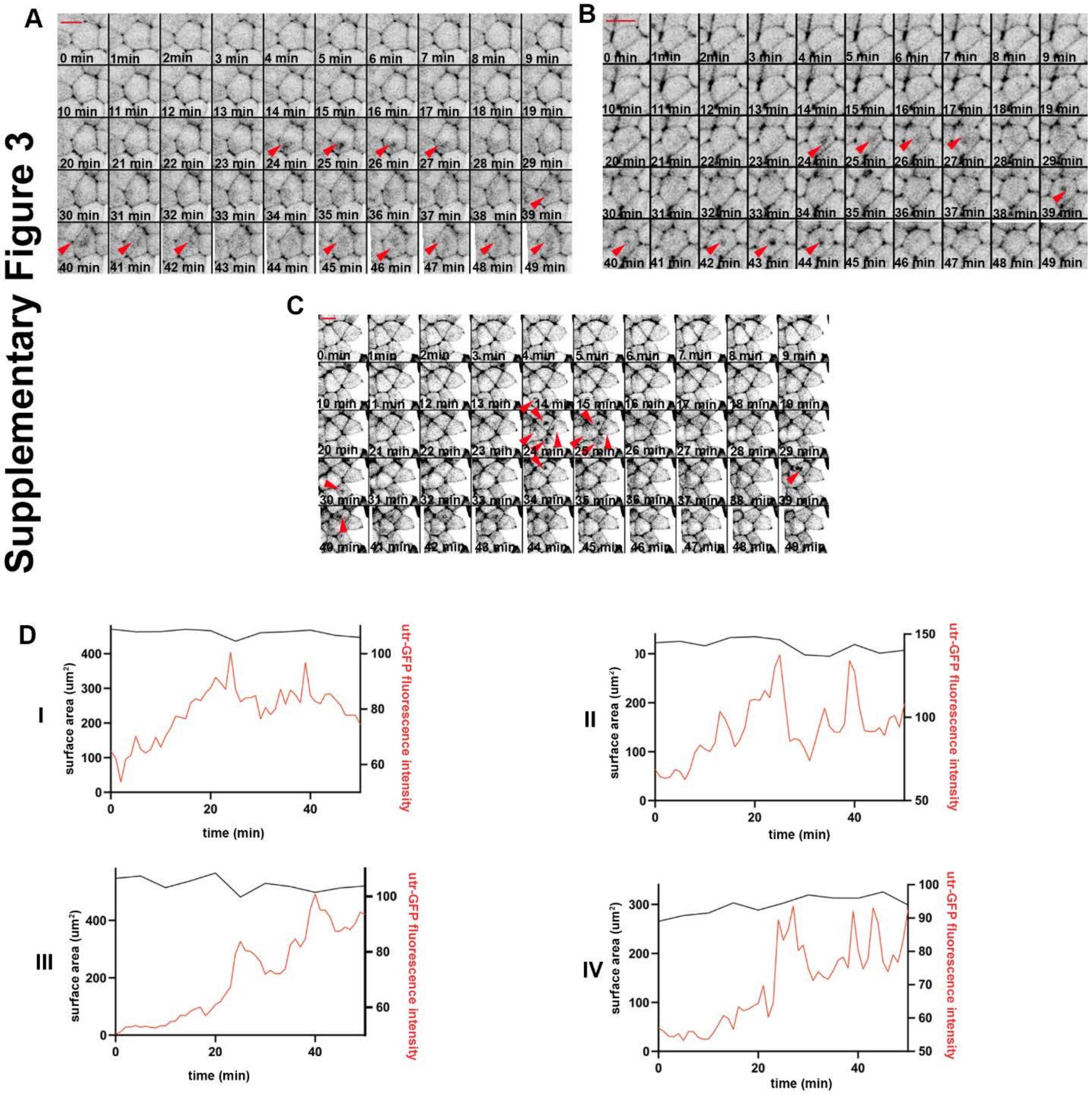

Supplementary Figure 3: Medioapical actin accumulation does not lead to reduction of the apical cell surface area of neuroepithelial cells overlying We2 morphant somitic mesoderm. A-C) Representative examples of neuroepithelial cells overlying Wee2 morphant somitic mesoderm. Red arrowheads: Apical F-actin accumulation**.** Scale bars: 20um **D)** Quantification of apical cell surface area (black line) and F-actin intensity (red line) over time in 4 representative neuroepithelial cells. F-actin accumulation is not followed by a decrease in apical cell surface area.

Supplementary Movies

Supplementary Movie 1: Somitic mesoderm morphogenesis influence neural tube closure. Neural tube close in an embryo with unilateral targeted somitic mesoderm Wee2 downregulation. Neural tube closure at the neuroepithelium overlying the morphant somitic mesoderm (left side) is defective. Anterior: top, Posterior bottom. Fluorescent marker: membrane GFP. Time interval= 3 min

Supplementary Movie 2. Defective somitic mesoderm morphogenesis results in impaired medial movement of neural plate/surface ectoderm boundary cells. Time lapse recording showing the movement of surface ectoderm cells found at the neural plate/surface ectoderm boundary. Surface ectoderm adjacent to the neural plate overlying normal somitic mesoderm (right side) moves towards the neural plate midline. The medial movement of the surface ectoderm adjacent to the neural plate overlying Wee2 morphant somitic mesoderm (left side) is defective. Anterior: top, Posterior bottom. Fluorescent marker: membrane GFP. Time interval= 3 min.

Supplementary Movie 3. Hinge point formation depends on somitic mesoderm morphogenesis. Time lapse recording of an embryo expressing the F-actin fluorescent marker Utrophin-GFP with unilateral somitic mesoderm knockdown of Wee2. Hinge point formation, evident by reduction of cell surface are and accumulation of F-actin, takes place normally at the neural plate side overlying control somitic mesoderm (right side). Hinge point formation is defective when the underlying somitic mesoderm morphogenesis is defective (left side). Time interval: 30 seconds.

Supplementary Movie 4. Defects in somitic mesoderm convergent extension are accompanied by defects in apical constriction within the neuroepithelium. Time lapse recording of an embryo expressing with unilateral somitic mesoderm targeted Wee2 knockdown. Apical constriction at the neural plate side (left side) overlying normal somitic mesoderm occurs normaly. Apical constriction is defective at the neural plate side (left) overlying Wee2 somitic mesoderm. Time interval 30 sec.

Supplementary Movie 5. Neighbor exchanges within the neural plate overlying somitic mesoderm displaying defective convergent extension. Two representative examples of polarized neighbor exchanges within the neuroepithelium overlying Wee2 morphant somitic mesdoderm. Time interval: 5 minutes.

Supplementary Movie 6. F-actin dynamics of control neuroepithelial cells undergoing apical constriction. Pulsed accumulation of medioapical F-actin is accompanied is accompanied by reduction of apical cell surface area. Time interval: 30 sec

Supplementary Movie 7. F-actin dynamics in neuroepithelial cells overlying Wee2 morphant somitic mesoderm. Pulsed medioapical F-actin accumulation is not followed by reduction of apical cell surface area. Time interval 30 sec.
